# Design programmes to maximise participant engagement: a predictive study of programme and participant characteristics associated with engagement in paediatric weight management

**DOI:** 10.1186/s12966-016-0399-1

**Published:** 2016-07-19

**Authors:** James Nobles, Claire Griffiths, Andy Pringle, Paul Gately

**Affiliations:** Institute of Sport, Physical Activity and Leisure, Centre of Active Lifestyles, Carnegie Faculty, Leeds Beckett University, Headingley Campus, Leeds, UK; MoreLife (UK) Ltd., Churchwood Hall, Leeds Beckett University, Headingley Campus, Leeds, UK

**Keywords:** Engagement, Attrition, Attendance, Paediatric, Family, Obesity, Weight management programme

## Abstract

**Background:**

Approximately 50 % of paediatric weight management (WM) programme attendees do not complete their respective programmes. High attrition rates compromise both programme effectiveness and cost-efficiency. Past research has examined pre-intervention participant characteristics associated with programme (non-)completion, however study samples are often small and not representative of multiple demographics. Moreover, the association between programme characteristics and participant engagement is not well known. This study examined participant and programme characteristics associated with engagement in a large, government funded, paediatric WM programme. Engagement was defined as the family’s level of participation in the WM programme.

**Methods:**

Secondary data analysis of 2948 participants (Age: 10.44 ± 2.80 years, BMI: 25.99 ± 5.79 kg/m^2^, Standardised BMI [BMI SDS]: 2.48 ± 0.87 units, White Ethnicity: 70.52 %) was undertaken. Participants attended a MoreLife programme (nationwide WM provider) between 2009 and 2014. Participants were classified into one of five engagement groups: Initiators, Late Dropouts, Low- or High- Sporadic Attenders, or Completers. Five binary multivariable logistic regression models were performed to identify participant (*n* = 11) and programmatic (*n* = 6) characteristics associated with an engagement group. Programme completion was classified as ≥70 % attendance.

**Results:**

Programme characteristics were stronger predictors of programme engagement than participant characteristics; particularly small group size, winter/autumn delivery periods and earlier programme years (proxy for scalability). Conversely, participant characteristics were weak predictors of programme engagement. Predictors varied between engagement groups (e.g. Completers, Initiators, Sporadic Attenders). 47.1 % of participants completed the MoreLife programme (mean attendance: 59.4 ± 26.7 %, mean BMI SDS change: -0.15 ± 0.22 units), and 21 % of those who signed onto the programme did not attend a session.

**Conclusions:**

As WM services scale up, the efficacy and fidelity of programmes may be reduced due to increased demand and lower financial resource. Further, limiting WM programme groups to no more than 20 participants could result in greater engagement. Baseline participant characteristics are poor and inconsistent predictors of programme engagement. Thus, future research should evaluate participant motives, expectations, and barriers to attending a WM programme to enhance our understanding of participant WM engagement. Finally, we suggest that session-by-session attendance is recorded as a minimum requirement to improve reporting transparency and enhance external validity of study findings.

**Electronic supplementary material:**

The online version of this article (doi:10.1186/s12966-016-0399-1) contains supplementary material, which is available to authorized users.

## Background

Despite one third of children in the UK having overweight or obesity [1], weight management (WM) programmes are only thought to serve between 0.5 % and 1.5 % of the childhood population with a weight issue [2]. Acknowledging that between 22-90 % of these children will continue to have obesity as an adult [3], and that obesity is strongly associated with a range of negative health conditions (e.g. type 2 diabetes, cardiovascular diseases, sleep apnoea, cancers and polycystic ovaries [4–7]), the need for effective WM programmes - which encourage strong participant engagement and demonstrate positive health-related improvement - is critical [8].

Participant dropout, and consequent programme attrition, challenges programme effectiveness, both in WM^13, 14^ and in chronic ill health management [9–13]. In paediatric WM alone, attrition ranges from 8-83 % [14, 15]. Higher participant attendance is associated with greater weight loss and health-related benefits in contrast to those with lower attendance or those who dropout [16, 17]. Of concern to policy makers and programme commissioners (those purchasing the programmes), dropout and attrition compromise the economic effectiveness of WM programmes. For the programme delivery team, dropout has shown to cause feelings of failure or that their efforts have had minimal impact [14, 18].

The majority of work into paediatric WM programme attrition (and WM programmes more broadly [19]) has reported predictors of dropout using pre-intervention participant characteristics (e.g. gender, age, and ethnicity) or reasoning for dropout via qualitative enquiry [14, 15]. Some studies have cited that youths with a higher body mass index (BMI) [20–22], of an ethnic minority [20, 23], and of low socioeconomic status (SES) [21, 24, 25] have a greater risk of not completing a programme. Furthermore, studies which have examined attrition often have small non-generalisable samples, with the majority of participants attending a clinical- rather than community- WM programme [26–28].

Non-standardised definitions and criteria for engagement-related terminology are also problematic and makes it exceptionally difficult, even impossible, to draw valid conclusions on the predictors of dropout and completion [14, 17]. Terms such as dropout and non-completion are used synonymously in the literature, but their definitions and criteria vary greatly [17]. A review identified the criterion for *dropout* in 23 paediatric WM studies [14]; criterion ranged from ‘did not complete study’ [27], to ‘attended ≤ 2 clinical appointments’ [26], to ‘did not return for follow-up visit after initial visit’ [29]. The same holds true for completion: inconsistent criteria for ‘completion’ make it difficult for policy makers to interpret programme outcomes (e.g. BMI reduction). Poor programme outcomes (e.g. no change/increase in BMI) can be masked by an undemanding completion criterion. As such, standardised definitions and criteria for engagement-related terminology have been called for to advance the transparent reporting of programme outcomes in WM and public health [14, 30].

The term engagement is characterised here as the family’s (i.e. minimum one parent/carer and child) level of participation in the WM programme, a definition adapted from Higher Education [31]. It is therefore only dependent on the measurement of one variable, participant attendance. Engagement is a broad term that encompasses various other sub-domains (e.g. completion and dropout), each of which are defined in Table 1. It is apparent in the literature that engagement is a complex phenomenon, and that multiple, clearly defined terms are required to describe a family’s engagement trajectory (i.e. to what extent they have engaged in the WMP) [14, 17, 20].

**Table 1:** Definition and criteria of the engagement groups

Engagement Group	Definition
Non-initiator	Enrol into a MoreLife programme but do not attend any sessions.
Initiator	Attend the first third of the programme only.
Late dropout	Attend the first and second third of the programme only.
Low sporadic attender	Attend <50 % of all sessions across the programme. Must attend at least one session in the middle and last third of the programme.
High sporadic attender	Attend between 50-70 % of sessions across all programme.
Completer	Attend ≥70 % of all sessions. Seventy percent was selected as it falls between the completion criterion recommended by the National Obesity Observatory [38] (75 %) and the Department of Health [39] (60 %).

This study sought to 1) investigate participant and programme characteristics which could predict engagement in a large, geographically diverse WM programme, 2) provide a plausible criterion that can be used to classify participant engagement, and 3) evidence programme effectiveness on BMI SDS change relative to engagement.

## Methods

### Study design and setting

Secondary analysis of data from MoreLife (UK) Ltd. (referred to as MoreLife) was undertaken. MoreLife delivers community-based, family WM programmes across the UK. For a family to be eligible to attend, their child must be classified with overweight or obesity; a BMI exceeding the 91^st^ centile (Standardised BMI [BMI SDS] ≥1.33 units) [32, 33]. MoreLife programmes in this study shared common characteristics: group-based format; free to attend (funded by the government); 10-12 weeks in length; weekly sessions of 2-3 h; standardised delivery protocol; focus on dietary advice, increasing habitual and structured physical activity and decreasing sedentary time; use behaviour change techniques; parents/carers attend alongside their children. Participants are predominantly recruited via self-referral, GP referral or via a school nurse. Key performance indicators and outcome measures are defined by the MoreLife programme commissioners (on behalf of the local government). A standardised programme description is provided in a supplement [34] (Additional file 1).

### Data

**Fig. 1:**
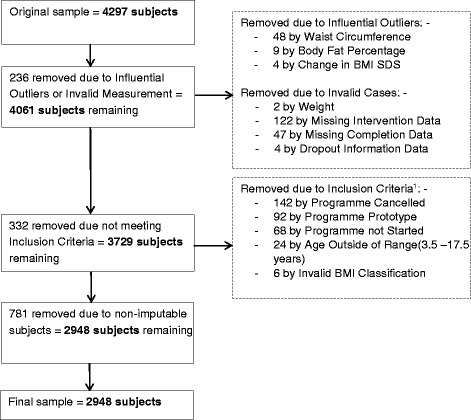
Data Exclusion and Validation. ^1^Inclusion Criteria: attend a 10-12 week, community-based, WMP; aged 3.5-17.5 years; classified with overweight or obesity

The initial data set included 4297 participants. Participants attended a programme between September 2009 and September 2014. Data were thoroughly examined against inclusion criteria and data validation processes were carried out (Fig. 1). A final sample of 2948 (68.6 %) participants from 244 delivered programmes was used. All of these participants attended one or more sessions of a MoreLife programme.

Missing data was problematic, ranging from 0.1 % missing (Age) to 54 % missing (Sedentary Behaviour). Multiple Imputation (MI) was used to maximise power and retain sample size. MI generates several imputed datasets based on the observed data, and analysis is carried out on each data set independently [35, 36]. A single estimate, and its associated standard errors, is finally generated by pooling the results of each imputed data set [35, 36]. Details of the data imputation process are given in a supplement using the Sterne Criteria [37] (Additional file 2).

Ethical approval was provided by Carnegie Faculty, Leeds Beckett University Research Ethics Committee (*ref*: 4869). All participants (and parents) gave consent for their data to be used for research purposes when starting the MoreLife programme.

### Participant engagement

MoreLife programme protocol stipulates that BMI is recorded weekly by a trained staff member if participants are present. Percentage of Attendance was subsequently calculated (attended sessions/available sessions X 100), and each participant was assigned to an engagement group (Table 1). Non-Initiators (*n* = 781) were initially to be included in analyses, however this subgroup had variables with >98 % missing data and were therefore removed from data set.

### Predictors of engagement

To explore the aims of the study, 17 independent variables were considered; 11 participant characteristics and six programme characteristics. Participant characteristics included: Age, Gender (male/female), Ethnicity (white/white British or non-white/non-white British), Indices of Multiple Deprivation (IMD) Score (proxy for SES; high scores indicate greater deprivation), Medical Conditions (yes/no [e.g. asthma, type 2 diabetes, dyspraxia, and autism]), Self-esteem (via modified Harter’s Self-Perception Profile [40]), Body Satisfaction (via Contour Figure Ratings Scale [41]) and Sedentary Behaviour (via modified version of Sedentary Behaviours Questionnaire [42]). Measures of BMI and waist circumference (WC) were standardised (SDS) using UK90 [31] and McCarthy et al. [43] reference data respectively. Clinical classifications (e.g. healthy weight, overweight) were applied to BMI SDS and WC SDS [32, 33, 43]. Programme characteristics consisted of: Programme Year, Group Size (≥20 participants or <20 participants), Age Groups (separated younger/older age or mixed age), Programme Length (10 weeks or 12 weeks), Day of Session Delivery (weekday or weekend), and Delivery Period (MoreLife programmes commence in either January, April, or September). Programme characteristics were generated by identifying elements of the protocol which vary between MoreLife programmes. Certain participant- and programme- characteristics had to be collapsed into two groups to enable the MI to be completed (e.g. Group Size, Ethnicity, and Medical Conditions).

### Statistical analysis

Descriptives (mean, SD) and frequencies (%) provided participant and programme characteristics. A one-way ANOVA test determined the reduction in BMI SDS between engagement groups, and a Pearson’s Correlation investigated the relationship between the Percentage of Attendance and Change in BMI SDS (difference between first and last measurement). Binary logistic regression models examined the inference of independent variables on numerous engagement group dichotomies. Initial univariable models (i.e. one independent variable and the dependent outcome) were completed before constructing a multivariable model. A multivariable model was developed systematically for Completers vs. Non-Completers; this dichotomy is commonly observed in the literature [14]. Four additional models were created using the same independent variables as the previous model, however engagement groups varied (Table 3). A forced entry method was applied in all multivariable models (i.e. all variables are conditional upon one another and none are removed). Odds Ratios (OR) and 95 % Confidence Intervals (CI) are presented for all models. Alpha set at 0.05.

## Results

### Participant engagement

**Fig. 2:**
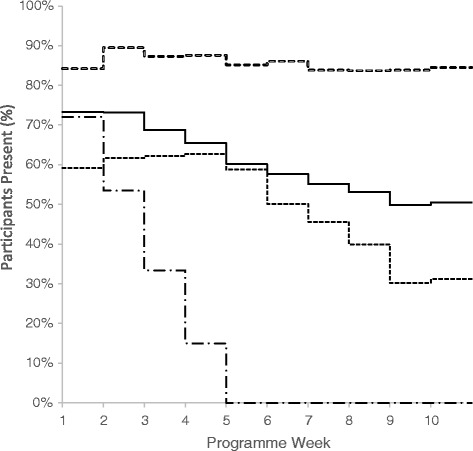
Weekly Session Attendance by Engagement Groups. The figure demonstrates the percentage of participants present each week. Weeks 11 and 12 are not displayed as MoreLife programmes do not all last 12 weeks. Solid line represents the sample as a whole (*n* = 2948), hollow-dashed line represents Completers (*n* = 1387), short-dashed line represents Non-Completers (*n* = 1013) and the long-dashed line represents Initiators (*n* = 548)

Participant characteristics are summarised in Table 2. Average participant attendance was 59.4 ± 26.7 %, and 47.1 % (*n* = 1387) of the sample completed (≥70 % attendance) the programme. The weekly attendance of the participants is displayed in Fig. 2 – engagement groups were collapsed to facilitate interpretation. Not all participants attended the first session, hence why Week 1 values lie between 59-84 %. Programme engagement reduced consistently when observing the whole sample (*n* = 2948).

**Table 2:** Participant characteristics

Characteristic	Mean or *n*	SD or %
Gender [*n*, %]		
Male	1340	45.45 %
Female	1608	54.55 %
Age (Years) [mean, SD]	10.44	2.80
Ethnicity [*n*, %]		
White/White British	2079	70.52 %
Non-white/Non-white British	869	29.48 %
IMD Score [mean, SD]	30.26	15.90
IMD Decile [*n*, %]^a^		
1 – Least deprived	64	2.17 %
2	101	3.43 %
3	129.8	4.40 %
4	201.3	6.83 %
5	190	6.45 %
6	293.2	9.95 %
7	406.6	13.79 %
8	464.3	15.75 %
9	573.1	19.44 %
10 – Most deprived	524.7	17.80 %
Medical condition [*n*, %]		
No	2727	92.50 %
Yes	221	7.50 %
BMI (kg/m^2^) [mean, SD]	25.99	5.79
BMI SDS (units) [mean, SD]	2.48	0.89
Waist circumference (cm) [mean, SD]	83.40	15.01
WC SDS (units) [mean, SD]	2.94	0.97
Obese or Non-obese [*n*, %]		
Obese	2161	73.30 %
Non-obese	787	26.70 %

^**a**^Decimal numbers reflect pooled estimate

### Predictors of engagement

The five multivariable models included consistent independent variables (to enable between-model comparisons), including: Ethnicity, IMD Score, BMI SDS, Programme Year, Group Size and Delivery Period. Other variables were not associated with programme engagement – all univariable results are provided in an online supplement (Additional file 3).

#### Model 1: Non-completion

The likelihood of not completing a programme (<70 % attendance) opposed to completing the programme (≥70 % attendance) was greater for those who attended a larger group (OR: 1.21, 95 % CI: 1.03, 1.42), attended in a recent programme year (OR: 1.13, 95 % CI: 1.07, 1.20), and started the programme in April (OR: 1.28, 95 % CI: 1.08, 1.53) or September (OR: 1.26, 95 % CI: 1.05, 1.52) rather than January. Higher IMD Score and higher BMI SDS also implied greater likelihood of Non-Completion – these associations were weaker than programmatic characteristics (Table 3).

**Table 3:** Predictors of engagement: multivariable results

	Multivariable Model Results		
	OR	95% Confidence Interval	*p*-value
Model 1: *Completer (n = 1387) vs. Non-Completion (n = 1561)*			
*Constant* ^a^	0.398	(0.278, 0.571)	<0.001
Ethnicity^b^	1.028	(0.835, 1.264)	0.80
IMD Score	1.005	(1.000, 1.010)	0.04
BMI SDS	1.111	(1.020, 1.211)	0.02
Programme Year	1.130	(1.066, 1.199)	<0.001
Group Size^b^	1.207	(1.028, 1.417)	0.02
Delivery Period^b^			
January Intake	*Reference Category*		
April Intake	1.284	(1.077, 1.530)	0.01
September Intake	1.261	(1.046, 1.520)	0.02
Model 2: *Continuer (n = 2400) vs. Initiator (n = 548)*			
*Constant*	0.079	(0.049, 0.127)	<0.001
Ethnicity^b^	0.637	(0.492, 0.825)	<0.001
IMD Score	1.003	(0.997, 1.009)	0.27
BMI SDS	1.091	(0.976, 1.220)	0.12
Programme Year	1.178	(1.090, 1.272)	<0.001
Group Size^b^	1.399	(1.141, 1.714)	<0.001
Delivery Period^b^			
January Intake	*Reference Category*		
April Intake	1.318	(1.054, 1.648)	0.02
September Intake	1.363	(1.069, 1.739)	<0.001
Model 3: *Completer (n = 1387) vs Late Dropout (n = 380)*			
*Constant*	0.097	(0.054, 0.173)	<0.001
Ethnicity^b^	0.823	(0.603, 1.125)	0.22
IMD Score	1.002	(0.994, 1.009)	0.64
BMI SDS	1.178	(1.023, 1.357)	0.02
Programme Year	1.167	(1.066, 1.278)	<0.001
Group Size^b^	0.957	(0.743, 1.232)	0.73
Delivery Period^b^			
January Intake	*Reference Category*		
April Intake	1.397	(1.064, 1.834)	0.02
September Intake	1.180	(0.874, 1.594)	0.28
Model 4: *Completer (n = 1378) vs. Sporadic Attender (n = 633)*			
*Constant*	0.214	(0.134, 0.343)	<0.001
Ethnicity^b^	1.565	(1.153, 2.124)	0.01
IMD Score	1.006	(1.000, 1.013)	0.04
BMI SDS	1.065	(0.949, 1.195)	0.28
Programme Year	1.032	(0.954, 1.117)	0.09
Group Size^b^	1.196	(0.970, 1.474)	0.09
Delivery Period^b^			
January Intake	*Reference Category*		
April Intake	1.123	(0.891, 1.414)	0.33
September Intake	1.165	(0.913, 1.485)	0.22
Model 5: *High- (n = 346) vs. Low- (n = 287) Sporadic Attender*			
*Constant*	0.510	(0.228, 1.144)	0.10
Ethnicity^b^	1.539	(0.980, 2.419)	0.06
IMD Score	1.010	(1.000, 1.021)	0.05
BMI SDS	0.863	(0.718, 1.038)	0.12
Programme Year	1.104	(0.956, 1.275)	0.18
Group Size^b^	1.358	(0.954, 1.933)	0.09
Delivery Period^b^			
January Intake	*Reference Category*		
April Intake	0.763	(0.517, 1.125)	0.17
September Intake	0.880	(0.583, 1.330)	0.55

^a^ *Constant* = Intercept of the Regression Line (β_0_)

^b^Categorical variables

Model 1: *R* ^2^ = 0.014-0.015 (Cox & Snell), 0.019-0.021 (Nagelkerke). Model χ^2^ (7) = 42.99-45.82

Model 2: *R* ^2^ = 0.013-0.017 (Cox & Snell), 0.021-0.027 (Nagelkerke). Model χ^2^ (7) = 39.18-50.00

Model 3: *R* ^2^ = 0.012-0.014 (Cox & Snell), 0.018-0.021 (Nagelkerke). Model χ^2^ (7) = 20.64-24.34

Model 4: *R* ^2^ = 0.011-0.022 (Cox & Snell), 0.015-0.031 (Nagelkerke). Model χ^2^ (7) = 22.22-45.11

Model 5: *R* ^2^ = 0.045-0.062 (Cox & Snell), 0.060-0.083 (Nagelkerke). Model χ^2^ (7) = 29.12-40.41

#### Model 2: Initiators

Participants of a non-white ethnicity were less likely to be an Initiator (attend in the first third of sessions only) than white participants (OR: 0.64, 95 % CI: 0.49, 0.83). Notably, participants in a larger group had 1.40 (95 % CI: 1.14, 1.71) times greater likelihood of only initiating the programme, and the likelihood of only initiating increased by 18 % (OR: 1.18, 95 % CI: 1.09, 1.27) each programme year (2009-2014). Participants starting the programme in April and September were also more likely to only initiate the programme.

#### Model 3: Late dropouts

Late Dropouts (attend in first and second third of the programme, do not attend in last third) were modelled against Completers. High BMI SDS, attending in more recent programme years and beginning a programme in April increased the likelihood of being a Late Dropout as opposed to a Completer; odds ratios of 1.18 (95 % CI: 1.02, 1.36), 1.17 (95 % CI: 1.07, 1.28) and 1.40 (95 % CI: 1.06, 1.83) respectively.

#### Models 4 & 5: Sporadic attenders

Model 4 grouped Sporadic Attenders together (Low and High Sporadic Attenders) against Completers. Non-white ethnicity (OR: 1.57, 95 % CI: 1.53, 2.12) and higher IMD Score (OR: 1.01, 95 % CI: 1.00, 1.01) increased the likelihood of sporadic attendance opposed to programme completion. Moreover, those of non-white ethnicity were 1.54 (95 % CI: 0.98, 2.42) times as likely as white participants to be a Low Sporadic Attender (attend <50 % across programme) than a High Sporadic Attender (attend between 50-70 % of programme). Per unit increase in IMD Score, the likelihood of being a Low Sporadic Attender was increased by 1 % (OR: 1.01, 95 % CI: 1.00, 1.02). Large group size suggested 1.36 (95 % CI: 0.95, 1.93) times greater likelihood of being a Low Sporadic Attender. Both this finding and that of ethnicity were not however, statistically significant.

### Change in BMI SDS

Completers achieved a BMI SDS reduction of 0.15 ± 0.22 units during the programme. Initiators, Late Dropouts, Low Sporadic Attenders and High Sporadic Attenders all demonstrated a reduction of 0.02 ± 0.20 units, 0.07 ± 0.21 units, 0.07 ± 0.18 units and 0.09 ± 0.18 units respectively. Completers elicited greater BMI SDS reductions than all other subgroups (*p* <0.001). Moreover, a weak negative correlation of -0.27 (*R*^*2*^ = 0.07*, p* <0.001) was observed between Percentage of Attendance and Change in BMI SDS.

## Discussion

This study found that participant characteristics were generally poor predictors of engagement, which disputes many past findings [14, 15]. Only BMI SDS, IMD Score and Ethnicity were observed to be inferential in some models – these were however weak predictors and should not be over-interpreted. Conversely, programmatic characteristics, particularly Group Size, Programme Year and Delivery Period, were stronger predictors of engagement. Participant and programme predictors varied between models which suggests that certain variables are more influential for different engagement groups – this has been observed elsewhere, specifically regarding participant-related characteristics [20, 23].

Six groups of participants were identified in this study by their programme engagement. The final analysis was conducted on five of the six groups after Non-Initiators were removed. Of all participants who signed onto the MoreLife programme (*n* = 3729), Non-Initiators made up 21 % (*n =* 781) of the sample, consistent with another study [24]. Perez et al. suggested that Non-Initiators may not have a perceived need for WM, or perhaps had no intention of participating when initially enrolling, whilst others may have barriers and situational factors that cause initiation to be postponed [44]. Given the costs associated with recruitment to WMPs and moreover, the cost associated with delivering WMPs, research is called upon to explore why one in five participants do not start the MoreLife programme. Future WMP teams could benefit from over-recruiting participants to anticipate that one fifth may not-initiate treatment (MoreLife currently utilise this approach).

### Predictors of engagement: *participant characteristics*

Studies have evidenced that older age [20, 21, 45], gender [29, 46, 47], and psychological status [28, 45, 46, 48] can predict dropout in paediatric WM programmes, yet this study showed that these characteristics were not statistically significant. Here, higher baseline BMI SDS was weak a predictor of Non-Completion, Initiation and Late Dropout. *Post-hoc* analysis revealed that Non-Completers had a 2 % higher BMI SDS than Completers (Non-Completers: 2.50 ± 0.0.90 units, Completers: 2.45 ± 0.86 units, *p* = 0.09).

IMD Score, a proxy measure for SES, was also a weak predictor: those living in an area of high deprivation had a small increased likelihood of Non-Completion, Sporadic Attendance and more specifically, Low Sporadic Attendance. Low SES has previously been a strong predictor of Non-Completion in North America [23, 29], however many American residents are required to pay or use health insurance to cover the cost of WM services. MoreLife programmes are government funded (i.e. free to attend), which perhaps explains why IMD Score is a weak predictor of engagement. That said, Fagg et al. found low SES to be an indicator of Non-Completion in a large UK-based study, speculating that families of lower SES had greater indirect relative costs associated with attending the programme (e.g. time and transport) [46].

Ethnicity was inconsistent within the regression models. Non-white participants were less likely than white participants to start the programme and subsequently dropout (i.e. initiate). Yet on the contrary, non-white participants were more likely to sporadically attend the programme as opposed to completing it. These findings are challenging to discuss. It would appear that participants of an ethnic minority are more likely to persist with a programme, but are not able to attend as consistently as white participants; non-white participants had 2.9 % lower attendance than white participants (white = 60.3 ± 29.7 %, non-white = 57.4 ± 28.3 %, *p* = 0.04). Cultural differences may be one explanation [18, 21, 25], but further research is required to explore differences in the role of WM services between ethnic groups [27].

### Predictors of engagement: *programme characteristics*

Large group size predicted poorer engagement in Models 1 (Non-Completers) and 2 (Initiators). Large group size was arbitrarily classified as ≥20 participants assigned to the programme - no known literature was available to guide this classification. Additional sensitivity analysis was undertaken which defined large group size as ≥10 and ≥15 participants; these group sizes were not shown to influence participant engagement. A recent paper of Odgers-Jewell et al. supports our findings, reporting that in groups of 4-16 participants, engagement was constant [49]. Our results, and those of Odgers-Jewell et al., would suggest that groups with fewer than 20 participants (at baseline) may have higher engagement than larger groups. Group programmes (opposed to one-to-one sessions) enable sessions to be more detailed, encouraging social interaction between peers and delivery staff, and for promoting a sense of group belonging rather than isolation [49].

The findings on group size may be useful to policy makers, who could improve the economic effectiveness of programmes by limiting programmes to no more than 20 participants. In a recent National Institute for Health and Care Excellence (NICE) costing report for designing childhood WM interventions [2], all programme costings were estimated based on a group size of 10 participants. The authors made group size vary by 50 % (5 and 15 participants) in a sensitivity analysis; the projected cost of delivering 2300 programmes in the UK differed by £6.4 million per year (£9.6 m and £3.2 m respectively). The findings from our paper would suggest a group size of approximately 20 participants is feasible to deliver and may also alleviate public health spending.

Programme Year was viewed as a proxy for programme fidelity in this study. In earlier years of the programme (2009-2011) there was a lower uptake of participants in contrast to later years (2012-2014); 881 and 2066 participants correspondingly. Participants who attended a programme in the earlier years, when fewer programmes were commissioned, had an average attendance of 63.2 ± 28.4 %. Compared to recent years, when the programme expanded, the average participant attendance was significantly lower at 57.8 ± 29.5 % (*p* <0.001). While this reduction in attendance may be due to scaling up of the programme, there is a dearth of research to support conclusive causal mechanisms [50]. Programme dissemination requires financial resources, staff training, management capabilities and infrastructure to be in place [30]; if programmes expand rapidly then the procedures outlined above may not be sufficiently established, leading to weaker/inconsistent outcomes [51, 52]. Evidencing the importance of financial resource, the funding per participant at MoreLife decreased by 37.5 % in recent years, however the efficacy of the programme was expected (by commissioners) to remain intact. A large reduction in funding may therefore explain poorer participant engagement in recent years.

Delivery period was the final programme characteristic to be associated with engagement. Mean participant attendance was 2.5-3 % lower between April-July in contrast to January-April and September-December. It may be viable to hypothesise that some participants would rather spend time outside recreationally than attend a programme indoors during the spring and summer months. This assumption lacks empirical evidence. Additionally, January experiences a surge in weight loss efforts after festive periods and commonplace weight gain [53].

### Programme engagement

Engagement in the MoreLife programme appears to reduce consistently (Fig. 2). A total of 2161 (73.3 %) attended Week 1, which gradually reduced to 1489 (50.5 %) in Week 10. A similar decline in programme engagement was reported by Dolinsky et al. [23]. Overall participant completion was also comparable with other studies [14, 15]; 1387 (47 %) attended ≥70 % of the available sessions. Average attendance amongst MoreLife participants was slightly lower than a similar scaled up programme, Go4Fun, based in Sydney, Australia (*n* = 2812*, n* programmes = 293) [30]. Go4Fun reported an average participant attendance of 64.5 % (54.1 % completion), while MoreLife an average of 59.4 % (BMI SDS change: Go4Fun; -0.11 units, MoreLife; -0.15 units). A year-by-year breakdown was not provided by Hardy et al. to evidence the impact of programme up scaling on fidelity and attendance [30].

### Terminology for engagement

Terminology and criteria for participant engagement are not standardised [17]. Should programme completion be defined in this study using an alternative criterion (e.g. ≥50 % attendance [54]), the percentage of Completers would invariably change (e.g. ≥50 % attendance = 67.3 % completion rate), as would the predictors of engagement [17]. As a result, our paper advocates widespread collection of session-by-session attendance data as a minimum requirement; this can then be translated into a percentage of attendance and subsequently used to classify programme engagement. This alone would lead to greater reporting transparency and clarity in engagement, attrition and more broadly, WM-related research [17, 30]. Whilst we would suggest that programme completion be defined as ≥70 % attendance, we understand that such a criterion is often pre-defined, especially within the UK, by programme commissioners and not the programme itself.

## Conclusion

This paper emphasises that WM programmes can be disseminated effectively, and that reduced attendance (in later years) may be the product of decreased financial resource and attenuated programme fidelity. Furthermore, WM programme providers may benefit from limiting group size to 20 participants and accounting for a 20 % non-initiation rate to maximise cost effectiveness. Research on programme engagement should move beyond the analysis of pre-intervention participant characteristics; all characteristics were shown to be weak predictors in this study. Miller, Brennan concluded that to advance in the field, predictors of engagement need to be planned a priori (i.e. barriers to treatment participation scale [55]) – a statement which we would advocate [17]. Finally, widespread collection of attendance data would improve reporting transparency and external validity [17]. Such conclusive remarks pose questions for future research.

## Abbreviations

BMI SDS, standardised body mass index; BMI, body mass index; CI, confidence intervals; IMD, indices of multiple deprivation; MI, multiple imputation; OR, odds ratio; SD, standard deviation; SES, socioeconomic status; TIDieR, template for intervention description and replication; UK, United Kingdom; WC SDS, standardised waist circumference; WC, waist circumference; WM, weight management

## Additional files

Additional file 1: TIDieR Framework: A Comprehensive Overview of the MoreLife Programme. (PDF 367 kb) Additional file 2: Treatment of Missing Data. (PDF 638 kb) Additional file 3: Univariable Regression Models. (PDF 411 kb)
