# Supplementary material for: Design programmes to maximise participant engagement: a predictive study of programme and participant characteristics associated with engagement in paediatric weight management
Source: Int J Behav Nutr Phys Act. 2016 Jul 19;13:76. doi: 10.1186/s12966-016-0399-1 (PMC4949908; doi:10.1186/s12966-016-0399-1)
Supplement: Additional file 1: — TIDieR Framework: A Comprehensive Overview of the MoreLife Programme. (PDF 367 kb) [file 12966_2016_399_MOESM1_ESM.pdf]

Additional File 1: *TIDieR Framework*

This supplement aims to provide the reader with an understanding of the MoreLife programme, beyond that described in the main paper itself. The TIDieR (Template for Intervention Description and Replication) has been adopted to detail 11 key domains of the programme design and delivery.

Programme Rationale

The MoreLife programme is a Tier Two programme as specified by the Obesity Care Pathway [1] – thus it is a non-pharmacological, non-surgical intervention aiming to reduce the degree of obesity through lifestyle modification. MoreLife has the intent of raising an awareness of obesity, and inspires action and offers treatment for childhood obesity [2]. The approach of the MoreLife is to deliver a programme grounded in research, and provide participants with techniques that are applicable in day-to-day living to evoke, small sustainable changes.

Given the prevalence of national childhood obesity, programmes such as MoreLife are warranted to educate and support healthy behaviour change in children (and families) with overweight or obesity. MoreLife, and other similar programmes, all form a part of the UK's public health strategy to stabilise and reduce the childhood obesity trends by 2020 [3].

Therefore, MoreLife programmes have a number of aims [4]: -

- Achieve a safe weight loss
- Reduce body fat
- Increase fitness
- Encourage positive self-worth

Programme Procedures

Referral: Participants are referred onto the programme through a number of avenues. These can include, but are not limited to, the following: self-referral, school-referral, professional-referral (GP, Dietician, Nurse etc...) and promotional events.

Participants are deemed applicable for the intervention if they have a BMI SDS $\geq$1.33 units; classifying the individual as above the 91st centile [5] and thus overweight using the clinical cut off points [6]. Some programmes use the 85th centile to classify overweight in children, the equivalent of a BMI SDS $\geq$1.04 units [5].

Pre-programme: All participants are sent a pre-MoreLife programme information pack. The families are made aware of the start date and the procedures to be anticipated in the first week.

Programme: MoreLife deliver a 10-12 week, community based WM programme for young people (4-17 years). One session of approximately two hours (some older programmes lasted up to three hours) is delivered per

week; these comprise of a physical activity element and a lifestyle education element. Programmes target the family unit, insofar that the parent(s)/carer(s) are expected to attend on a weekly basis.

BMI is recorded weekly and WC monthly. Psychological and behavioural questionnaires (Self-esteem, Body Satisfaction, and Sedentary Behaviour) are conducted pre and post intervention.

Maintenance: Families receive support for three months post intervention. The intensity of the support is dependent on the specifications of the commissioners, however frequently includes phone calls, text messages and emails. Unlimited access to a member's only website is given. Optional weekly drop in sessions are occasionally provided by certain programmes – this differs based on programme commissioner requirements.

Follow Up: MoreLife does not specifically collect follow up data.

Materials Used

All participants receive a host of MoreLife materials including a programme handbook. Additional incentives are given throughout the programme such as pedometers, water bottles, bags and t-shirts.

Staff refer to a curriculum (protocol) for the delivery content and structure of each session - this helps ensure a standardised programme delivery with strong fidelity.

Programme Provider

MoreLife is a limited (Ltd.) company, one which is a subsidiary company of Leeds Beckett University. Although centrally managed in Leeds, the company has management hubs in London, Oxford, Essex and the North East of England.

The programme has been designed and developed by a multi-disciplinary team at MoreLife. The team consists of experts in obesity, nutrition/dietetics, psychology and physical activity. As MoreLife is a subsidiary company of Leeds Beckett University, they share several joint appointments to ensure an applied research approach.

Programmes are delivered by staff with a strong interest and expertise in health, fitness and wellbeing.

Delivery staff are trained about the MoreLife curriculum over a number of days. This includes demonstration of ideal sessions, how to respond in difficult scenarios and how to implement a MoreLife programme as per the protocol. The aim is to ensure staff are confident and competent in programme format and delivery.

Mode of Delivery

The MoreLife community programme is delivered in a group based format. Group size varies dependent on commissioner specification and participant demand for the programme. As such, groups can range from 1-25 participants (*mean*: 12 participants). Parents and children are encouraged to take part in both physical activity and lifestyle elements each week.

Programme Location

Programme location varies dependent on the local government authority area health services purchasing MoreLife. MoreLife currently delivers programmes within 14 local government authorities across the UK. The

volume of delivery varies by programme location; one area completed 55 of the MoreLife programmes, whilst another has completed five programmes. Programmes per area vary between these two parameters.

Sessions typically run on a weekday evening, between 4 and 8 pm, or alternatively on a weekend. This varies by area, commissioner preference and venue availability.

Many of the areas worked within have been prioritised for health improvement by local authorities, and specifically WM services. What's more, programmes are often delivered in areas of low Socioeconomic Status (See Table 2: Main Study).

Programme Frequency/Duration

The programmes are 10-12 weeks in length, with weekly sessions lasting 2-3 hours. Sessions are broken down as discussed in criteria #4: *Programme Procedures*. Many of the frequency/duration aspects are specified by the programme commissioners but do not vary beyond the limits outlined above.

Tailoring

Although participants attend a group based programme, they are offered a weekly opportunity to discuss with a staff member concerns they may have as a family.

Families are encouraged to set long-term goals and weekly short term goals throughout the programme. Goals are set by the family, and are specific to their lifestyles.

Finally, programmes may be modified to cater for different sub groups, more so when a programme has a high percentage of non-white Caucasian participants. As such, sessions are tailored to be culturally specific.

Programme Modifications

Major Modifications: Redevelopment of the programme curriculum occurred in 2012. This was in response to the establishment of six new MoreLife delivery areas. The new curriculum intended to standardise programme delivery and ensure an exact methodology was replicated across all delivery areas. Session content and objectives were clarified in this curriculum update.

Additionally, MoreLife rebranded in July 2012. Prior to 2012 the company was known as Carnegie Weight Management and briefly CWM Health. Rebranding was across all MoreLife materials (Parent and Child Handbooks, Posters, Water Bottles etc…).

Minor Modifications: Staff changes occurred throughout delivery periods in most areas. Additionally, certain areas refined programme content for Black and Minority Ethnic groups to improve engagement.

Central London programmes required a reallocation of finances. Venues and transportation costs accounted for a greater proportion of the programme budget than programmes outside of London (e.g. North of England).

Programme Reporting and Fidelity

Staff members are encouraged to adhere to the MoreLife curriculum to ensure that a standardised programme is delivered in, and between, areas. That said, how strong the programme fidelity is differs due to commissioner

requirements, participant preferences, staff expertise and competencies. Additionally, although each of the 14 local areas is managed by a senior programme manager, multiple programmes are running concurrently which can make the assessment and maintenance of (strong) programme fidelity difficult.

Delivery staff members are encouraged to provide feedback on session delivery, and discuss any issues encountered with programme co-ordinators. Programme co-ordinators and programme managers can escalate messages/queries to the central, senior management team. How far programme fidelity is met across areas remains somewhat unknown, however it is centrally promoted, managed and evaluated by the senior management team.

References:

1. Department of Health. *Developing a specification for lifestyle weight management services: best practice guidance for tier 2 services*. In: Department of Health (ed). Department of Health: London, UK, 2013. 1-42.

2. MoreLife. *About us*: MoreLife; 2015a [cited 2015 16/03/2015]. Available from: http://www.more-life.co.uk/Default.aspx?PageName=About.

3. Department of Health. *Healthy lives, healthy people: a call to action on obesity in England*. In: Department of Health (ed). Department of Health: London, UK, 2011. 1-54.

4. MoreLife. Family clubs: Does club work? : MoreLife; 2015b [cited 2015 16/03/2015]. Available from: http://www.more-life.co.uk/Default.aspx?PageName=ServiceClub.

5. Cole TJ, Freeman JV, Preece MA. Body Mass Index reference curves for the UK, 1990. *Arch Dis Child.* 1995;**73**: 25-29.

6. Dinsdale H, Ridler C, Ells LJ. *A simple guide to classifying Body Mass Index in children*. In: National Obesity Observatory (ed). National Obesity Observatory: Oxford, 2011.
