# Supplementary material for: Design programmes to maximise participant engagement: a predictive study of programme and participant characteristics associated with engagement in paediatric weight management
Source: Int J Behav Nutr Phys Act. 2016 Jul 19;13:76. doi: 10.1186/s12966-016-0399-1 (PMC4949908; doi:10.1186/s12966-016-0399-1)
Supplement: Additional file 2: — Treatment of Missing Data. (PDF 638 kb) [file 12966_2016_399_MOESM2_ESM.pdf]

__Additional File 2__: ***Treatment of Missing Data***

For the vast majority of quantitative researchers, missing data are a problematic issue. Improper handling of missing data can lead to skewed and biased results which may not reflect the actual sample that has been subject to testing or observation [1]. In addition, complete case analyses (participants with no missing data) can reduce the standard error of the outcome, and moreover distort regression coefficients, such as those used within this study [1]. The reason for missing data varies between studies, but it is of utmost importance to understand the mechanisms leading to missing data (known as the Missingness Mechanism). This consideration is often overlooked by workers in the field, who may rely solely on the analysis of complete case data [2].

The following supplement outlines the approach taken to deal with missing data using the reporting guidelines of Sterne *et al.* (2009). The criteria comprises of 14 items, and has been adopted within similar studies in this field [3].

Criteria Item #1: *Report the number of Missing Values for Each Variable of Interest*

Of the variables included within the analysis, 13/20 were identified to have a degree of missingness, as demonstrated below in Table 1. Some of these variables, such as IMD, are derived from postcode (UK equivalent of ZIP Code) identifiers and thus the same level of missingness is evident across the range of postcode related variables. The level of missingness is shown to range from as little as 0.10% in Age through to 53.70% in Sedentary Behaviour. The reasoning for varying levels of missingness is later explained. However, the level of missingness across the variables is substantial enough to warrant further investigation and be subject to a form of data imputation [2].

Table 1: Degree of Missingness by Variables

**Variable**[1]	**Missing Data**	
	n	*%*
Sedentary Behaviour	2003	53.70%
Self-esteem	1465	39.30%
Ethnicity	1341	36.00%
Body Satisfaction	1258	33.70%
Body Fat Percentage	1142	30.60%
Standardised Waist Circumference	1116	29.90%
Waist Circumference	1098	29.40%

BMI Classification	818	21.90%
BMI SDS	818	21.90%
IMD Variables (Score, Rank, Decile)	161	4.30%
Age	4	0.10%

[1]Only variables with a degree of missingness are presented.

Criteria Item #2: *Reasoning for Missing Values*

There are 20 variables of interest used within this study, 13 of which have a degree of missingness. A number of variables (e.g. gender, medical conditions…) had complete data – the reasoning for this was due in part to data collection protocol of MoreLife: certain variables were mandatory to be completed by the participants before enrolling on the programme. Moreover, programme specific variables were complete as they are derived from the characteristics of the programme themselves, and are not affected by participant completion of the pre-entry documentation.

Where variables have missing data (Table 1), it is not possible to state why for each individual participant (*n* = 3729), instead it is possible to interrogate the dataset as a whole and further, split by programme location to uncover trends in missingness. The trends in this data set suggest that data are missing systematically among some of the programme locations - this has also been confirmed by the data provider, MoreLife. Although MoreLife collect data in accordance to a protocol, different programme delivery areas are managed by various teams, and overseen by programme commissioners. As a result, these commissioners are able to select which measures they wish to collect data on, hence the reason why Sedentary Behaviour is missing data in 54% of cases. The missingness is therefore not due to the participants, but to the programme which they attended (Table 2). As such, it is unlikely that cases with missing data differ significantly from those with complete cases and so imputation is a plausible method to counter missing data.

Table 2: Participants with Complete Data by Programme Location

**Area**	**Participants with Complete Data**	
	n	%
Berkshire	41	34.70%
Bexley	78	59.50%
Camden	37	33.33%
Doncaster	165	31.98%
Islington	133	50.40%
Knowsley	14	7.22%

Essex	49	15.96%
Middlesbrough	7	3.98%
North Yorkshire	12	24.49%
Peterborough	137	37.23%
Rotherham	0	0.00%
Redcar & Cleveland	14	10.45%
Stockton	15	6.85%
Wandsworth	207	49.40%

Dependent on Total Sample (*n* = 2948)

On further inspection, many of the data appear to be missing due to participant's classified as Non-Initiators. Non-Initiators sign on to the programme, and in doing so, provide some basic information (e.g. gender, age, medical condition), however they do not attend any of the sessions within the programme. The MoreLife protocol stipulates that anthropometric measures and questionnaires are completed in the first week, but as Non-Initiators do not attend the first week, they do not have any of these measures recorded. It is of utmost importance when working with missing data to identify if the missingness can be explained by observed data in the sample. It is fair to conclude that the missingness can be explained by the two variables discussed: Completion Status and Programme Area.

Little's MCAR test was implemented to establish if data are missing completely at random (MCAR). The result inferred they are not MCAR due to a significant chi-squared value ($\chi^2 = 868.98$, $df = 300$, $p = 0.00$). This would suggest data are either Missing at Random (MAR) or Missing Not at Random (MNAR). While Missing Value Patterns (Figure 1) can depict and demonstrate patterns in the missingness amongst all the variables – this alone does not enable one to assume why data are missing. Monotonicity (an increasing or decreasing pattern, with blocks of missing data in the lower right of the graph and blocks of complete data in the upper left) in the Missing Value Patterns was observed, as there were clear patterns in the figure – specifically identifying a positive monotonic pattern (upward steps). Table 3 provides ancillary information to Figure 1 by identifying which 10 patterns of missingness are most prevalent across all cases. As shown, Ethnicity alone is missing in 20.43% of all cases, whilst the combination of Ethnicity and Sedentary Behaviour is missing in 2.86% of all cases. It is possible to interpret Figure 1 in both a horizontal and a vertical manner; horizontally one can identify the pattern of missingness, whilst vertically one can quantify on how many occasions a variable was missing (e.g. Sedentary Behaviour was missing in 62.5% of all patterns).

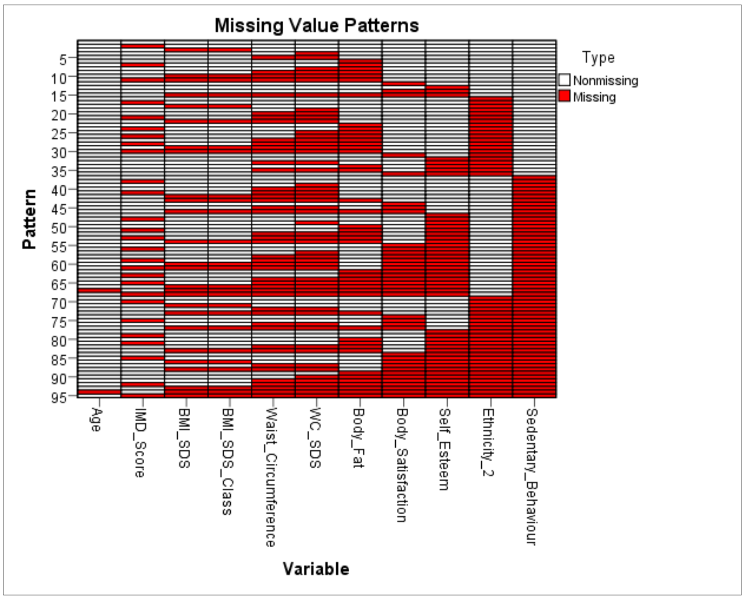

Figure 1: Missing Value Pattern in Data

Table 3: Prevalence of Missing Data Patterns

Pattern	Prevalent in Cases (%)	Variables Missing
1	33.96%	None
16	20.43%	Ethnicity
66	12.53%	BMI SDS, BMI Class, WC, WC SDS, BF, Body Satisfaction, Self-esteem and Sedentary Behaviour
93	9.87%	BMI SDS, BMI Class, WC, WC SDS, BF, Body Satisfaction, Self-esteem, Ethnicity and Sedentary Behaviour
37	8.24%	Sedentary Behaviour
55	6.43%	Body Satisfaction, Self-esteem and Sedentary Behaviour
69	2.86%	Ethnicity and Sedentary Behaviour
84	2.36%	Body Satisfaction, Self-esteem, Ethnicity and Sedentary Behaviour
50	1.72%	Body Fat, Self-esteem and Sedentary Behaviour
80	1.60%	Body Fat, Self-esteem, Ethnicity and Sedentary Behaviour

Data were assumed to be MAR. The assumption of it being MCAR was refuted with the result of Little's MCAR test. Data were also not assumed to be MNAR because the missingness could be rationally and completely explained by observed data [2]. From this point forth, data are treat as MAR.

Criteria Item #3: *Removal of Data due to Missing Values*

Given that data are assumed to be MAR, a number of processes need implementing before data are considered suitable for any form of data filling. Part of this process is to remove data according to an exclusion criteria (See Figure 1: Main Study). Aside data exclusion discussed previously, an additional removal criterion was applied to the sample before it was fit for data filling. This will be expanded upon here.

Briefly, data were removed due to Influential Outliers, Invalid Measurements, and participants not meeting the Inclusion Criteria. The initial sample of 4297 participants was therefore reduced to 3729 participants (86.8% of original sample) in the first phase of data exclusion (Figure 1: Main Study).

Within the Sterne *et al.* (2009) criteria Item 2, one particular group of participants were highlighted to have a vast proportion of missing data, Non-Initiators. Table 4 demonstrates the level of missingness amongst variables when split by completion status and as observed, missingness reaches almost 98% in one of the variables. A further six variables had missingness greater than 90%. It is not possible to gather any statistically meaningful results from a group of participants with minimal data, and as a result Non-Initiators (*n* = 781) were removed from the sample.

The initial sample of participants was reduced to a final figure of 2948 participants. This equates to 68.6% of the initial sample. A number of options are available for countering the issue of missing data, one of which is to use data from participants with complete cases. If such an analysis was to be conducted, data of 907 participants (30.8%) would be eligible for use. This approach, known as *list wise deletion*, has been advocated when missingness is present in less than 5% of cases [4]. Here, with missingness in 69.3% of the participants' cases, this approach would not be a suitable, ethical or valid method of dealing with missing data.

A total of 1349 participants were removed from the sample (see Figure 1). All future analysis will be conducted using the remainder of the initial sample (*n* = 2948).

Table 4: Data Missingness by Completion Status

Variable	Non-Initiator (n = 781)		Initiator (n = 548)		Non-Completer (n = 380)		High IA[a] (n = 346)		Low IA[a] (n = 287)		Completer (n = 1387)	
	n	%	*n*	%	*n*	%	*n*	%	*n*	%	*n*	%
Age (Years)	0	0.00%	0	0.00%	0	0.00%	0	0.00%	0	0.00%	4	0.29%
BMI Classification	741	94.88%	26	4.74%	6	1.58%	2	0.58%	11	3.83%	32	2.31%
BMI SDS	741	94.88%	26	4.74%	6	1.58%	2	0.58%	11	3.83%	32	2.31%
Body Fat Percentage	765	97.95%	110	20.07%	52	13.68%	18	5.20%	38	13.24%	159	11.46%
Body Satisfaction	718	91.93%	164	29.93%	71	18.68%	53	15.32%	72	25.09%	180	12.98%
Ethnicity	325	71.27%	198	36.13%	144	37.89%	123	35.55%	104	36.24%	447	32.23%
IMD Decile	29	3.71%	28	5.11%	10	2.63%	14	4.05%	22	7.67%	58	4.18%
IMD Rank	29	3.71%	28	5.11%	10	2.63%	14	4.05%	22	7.67%	58	4.18%
IMD Score	29	3.71%	28	5.11%	10	2.63%	14	4.05%	22	7.67%	58	4.18%
Sedentary Behaviour	747	95.65%	280	51.09%	154	40.53%	125	36.13%	115	40.07%	582	41.96%
Self-esteem	738	94.49%	198	36.13%	106	27.89%	64	18.50%	83	28.92%	276	19.90%
WC	748	95.77%	137	25.00%	53	13.95%	15	4.34%	41	14.29%	104	7.50%
WC SDS	748	95.77%	139	25.36%	59	15.53%	19	5.49%	45	15.68%	104	7.50%

[a]*IA - Infrequent Attender*

Criteria Item #4: *Differences between Complete and Incomplete Cases*

When working with missing data, it is important to distinguish differences between participants with complete cases and those with missing cases, especially with regards to participant characteristics. This enables one to observe if analysis on complete data may underestimate, and thus not provide a representative picture, the characteristics of the total population. Therefore, all participant variables were assessed for differences.

Table 5 and Table 6 demonstrate that significant differences were present between participants with complete and incomplete data. These differences included: Ethnicity, IMD variables, Medical Conditions, Age, Attendance and Completion Status, and Waist Circumference. BMI Classification, BMI SDS, Sedentary Behaviour and Gender were marginally deemed insignificant – this suggests that a degree of difference was present, although not substantial enough to provide statistical significance. There were no significant differences between Self-esteem and Body Satisfaction.

These findings are substantive, insofar that if analysis was to be conducted solely based on the participants with complete data, then the findings may be biased – particularly where significant differences were found between complete and incomplete cases. This reassures the need for an approach to filling missing data.

Table 5: Differences in Participants with Complete Data (*n* = 906) and Incomplete Data (*n* = 2042): Categorical Variables

Variable	Complete (*n* = 906)		Incomplete (*n* = 2042)		χ^2
	n	%	*n*	%	*p-value*
Gender					*0.054*
Male	388	42.8%	952	46.6%	
Female	518	57.2%	1090	53.4%	
Completion Status					*<0.001*
Completer	462	51.0%	925	45.3%	
Non-Completer	225	24.8%	703	34.4%	
Infrequent Attender	219	24.2%	414	20.3%	
Ethnicity					*<0.001*
White/White British	480	53.0%	828	80.7%	
Non-white/Non-white British	426	47.0%	198	19.3%	
BMI Classification					*0.065*
Severely Obese	378	41.7%	908	46.2%	

Obese	267	29.5%	564	28.7%	
Overweight	170	18.8%	339	17.3%	
Healthy Weight	91	10.0%	154	7.8%	
IMD Decile					*<0.001*
1 - Least Deprived	24	2.6%	40	2.1%	
2	30	3.3%	71	3.7%	
3	30	3.3%	99	5.2%	
4	72	7.9%	126	6.6%	
5	59	6.5%	118	6.2%	
6	89	9.8%	179	9.4%	
7	117	12.9%	255	13.4%	
8	167	18.4%	266	13.9%	
9	203	22.4%	352	18.4%	
10 - Most Deprived	115	12.7%	404	21.2%	
Pre-Existing Med Conditions					*0.035*
Yes	54	6.0%	167	8.2%	
No	852	94.0%	1875	91.8%	

Table 6: Differences in Participants with Complete Data (*n* = 906) and Incomplete Data (*n* = 2042): Continuous Variables

**Variable**	**Complete** (*n* = 906)		**Incomplete** (*n* = 2042)		
	Mean	*SD*	*Mean*	*SD*	*p-value*
Attendance (%)	0.64	0.27	0.57	0.30	<0.001
Age	10.21	2.79	10.55	2.81	0.003
IMD Score	28.69	14.05	31.04	16.89	<0.001
BMI	25.64	5.83	26.15	5.68	0.026
BMI SDS	2.44	0.90	2.50	0.86	0.077
Self-esteem	4.16	1.18	4.09	1.20	0.161
Sedentary Behaviour	3.38	1.85	3.56	1.83	0.054
Body Satisfaction	28.42	19.71	29.28	18.70	0.287
Waist Circumference	82.35	13.92	83.83	14.42	0.011
WC SDS	2.94	0.86	2.92	0.92	0.601
Body Fat (%)	35.12	8.56	35.25	8.31	0.717

Criteria Item #5: *Analysis used for Missing Data*

Data were assumed to be MAR, the rationale for which was explained in Criteria Item #2. Multiple Imputation (MI) was employed within the study to counter the issue of missing data. MI make use of observed data (i.e. cases with data), and its distribution, to predict what the missing value may be –

however, in order to ensure that the standard errors and variance are not subject to shrinkage or overstated precision, multiple data sets are created. The recommended number of imputed data sets is between 3-10, dependent on sample size, included variables and volume of missing data [5, 1, 2]. Imputing multiple data sets allows for uncertainty about missing data [2]: different simulated values will be imputed in each new data set (i.e. missing data are replaced with plausible, simulated values). Multiple data sets allow within-imputation (i.e. uncertainty in the estimate of imputed value) and between-imputation (i.e. uncertainty between imputed data sets) variability [6, 7]. MI consequently produces statistically unbiased estimates and standard errors [1]. After multiple data sets are formed and missing data are imputed, analysis of data are performed as normal (e.g. Regression, Correlation, Difference Testing etc...). Many statistical packages (SPSS, SAS, and Stata) will allow the conventional analysis of data after imputation, and outputs are subsequently provided for each of the imputed data sets. Furthermore, using Rubin's Rules [8] the outputs from each data sets are merged to form one 'pooled' estimate and its standard error. It is the pooled estimates which are reported in the literature and in this study.

This plausible method of dealing with missing data have been previously used in the area of engagement research [3] and the wider health related research [7, 9]. Other methods are available when working with missing data, however MI has been adopted due to its frequent use in similar studies (the paper of Hayati Rezvan *et al*. identified 103 studies utilising MI) and the relevance of its use in this scenario.

Criteria Item #6: *Software Used and Key Settings for Imputation Model*

Data were imputed using the Statistical Package for the Social Science (SPSS), version 21 (SPSS INC, Chicago, IL). The potential to undertake MI using SPSS was introduced in a recent development of the software, and is becoming more widely used in the field [7]. Other programmes such as SAS and Stata can also be used for MI.

A fully conditional specification (Multiple Imputation by Chained Equations) was used to impute data. This specification utilises sequential regression to impute the missing values dependent on the additional, specified variables in the model. For example, three variables may have missing data within them, and the missingness mechanism suggests data are absent due to two of the other, complete variables. A fully conditional specification accounts for the variables which can explain the missingness mechanism. This will be discussed further in the next item.

The fully conditional specification is also able to work with multiple data types (continuous, nominal and interval). This is possible as each variable included in the model is imputed using its own model [1]. SPSS enables the researcher to define the parameters of each variable to ensure the imputed values are consistently classified (e.g. an imputation for Gender [Coded 0 & 1], would only impute a value of either 0 or 1, as defined by the researcher – this prevents values such as 0.27 or 0.66 [for example only] being imputed).

Criteria Item #7: *Number of Imputed Data Sets Generated*

Ten data sets were imputed in order to reduce the sampling variability from the imputation process. Although five data sets are recommended by Sterne et al. (2009), by enabling 10 data sets to be imputed, the sampling variability (i.e. the amount of variation in the distribution of observed data) will have a lower variability and be theoretically, a more precise estimate.

The imputation conducted used a maximum of five parameter draws and a maximum of 500 case draws. A 2500 iteration model was therefore performed, 250 iterations for each of the 10 imputed data sets. When data are imputed, a number of plausible values are tested (one value per iteration of the model), and at each step of the iteration another missing value is completed whilst accounting for the first imputed value. This process is cyclic and as such requires many iterations [1].

Many of the variables included in the MI model were present as predictor variables only (with complete data). By including the dependent, outcome variables (Attendance and Completion Status) in the model, it ensured that these variables are accounted for because they hold certain information about the missing values. If attendance were omitted from the model, then this variable would not be accounted for when data are imputed. This can lead to biased and improper associations between the predictor variables and the dependent, outcome variables [2].

For sensitivity analysis, models with fewer iterations were completed (e.g. 5 data sets, 500 case draws and 5 parameter draws), but this did not yield differences between the descriptive statistics. Table 7 outlines the differences between the descriptive statistics when implementing different imputation methods and including a variety of predictor variables. The larger case draw (2500 iteration model) was taken forth as the imputed data set (Labelled *Final* in Table 7).

Table 7: Sensitivity Analysis of Imputation Models[a]

Variable	LW Deletion	V1	V2	V3	V4	V5	V6	Final
Attendance	0.00	0.00	0.00	0.00	0.00	0.00	0.00	0.00
Age	-0.01	0.00	0.00	0.00	0.00	0.00	0.00	0.00
IMD Score	0.00	-0.03	0.03	-0.02	-0.02	0.00	0.02	0.00
IMD Decile	0.00	0.00	0.00	0.00	0.00	0.00	0.00	0.00
BMI SDS	0.00	-0.01	-0.01	-0.01	-0.01	-0.01	-0.01	-0.01
Self-esteem	0.00	0.00	-0.01	-0.01	-0.01	-0.01	-0.01	-0.01
Sedentary Behaviour	0.00	0.13	0.16	0.16	0.15	0.07	0.06	0.06
Body Satisfaction	-0.03	-0.22	-0.24	-0.08	-0.25	-0.23	-0.20	-0.19
WC SDS	0.00	0.00	0.00	0.00	0.00	0.01	0.01	0.01
Body Fat %	0.01	-0.06	-0.07	-0.05	-0.06	-0.06	-0.05	0.00
Ethnicity	-0.10	-0.60	-0.10	-0.90	-0.17	3.17	2.81	2.80
BMI Classification	-0.10	-0.60	-0.40	-0.35	-0.57	-0.48	-0.48	-0.39

[a]Results demonstrate the difference between the original (non-imputed) and the imputed data. Units are relative to each variable.
LW: Listwise Deletion
V1: 2500 iteration model: 10 data sets, 500 case draws, 5 parameter draws.
V2: 2500 iteration model: 5 data sets, 500 case draws, 5 parameter draws.
V3: 1250 iteration model: 10 data sets, 250 case draws, 5 parameter draws.
V4: 500 iteration model: 10 data sets, 250 case draws, 5 parameter draws.
V5: 2500 iteration model: 10 data sets, 500 case draws, 5 parameter draws.
V6: 2500 iteration model: 10 data sets, 500 case draws, 5 parameter draws.
Final: 2500 iteration model: 10 data sets, 500 case draws, 5 parameter draws.

Criteria Item #8: *Variables Included in the Final Imputation Model*

SPSS (SPSS INC, Chicago, IL) was used for the purpose of MI. A total of 21 variables were entered into the imputation model which would then be accounted for when imputing missing data (Table 8). *Predictor Only* variables were those which were completely observed and had no values missing. More importantly, a number of these variables help to explain the missingness mechanism and are therefore required to impute missing data. Of utmost importance is the inclusion of the outcome variables: Completion Status and Percentage of Attendance. Not including the outcome variable could weaken the associations with the predictor variables [2]. *Partially observed* variables were those with a proportion of missingness. These variables were still used by the MI model to impute missing values in other variables and in their own variable. All participant related variables were included in the MI model to facilitate the most reliable imputation of missing data.

For continuous variables with missing data, SPSS allows the researcher to define the upper and lower parameters. This ensures that any imputed values fall within a specified range. The MI process makes it unlikely that the imputed, missing values will exceed the parameter limits. To impute

categorical data, all variables had to be binary coded – this required some variables to be collapsed. This will be discussed in the criteria item #9.

Table 8: Variables in the Imputation

Variable	Data Type
Predictor Only Variables (i.e. Completely Observed)	
Completion Status	Unordered Categorical
Percentage of Attendance	Continuous
Area ID	Unordered Categorical
Intervention ID	Unordered Categorical
Gender	Unordered Categorical
Age	Continuous
Medical Condition	Ordered Categorical
Predictor and Imputed Variables (i.e. Partially Observed and Require Imputing)	
IMD Score	Continuous
IMD Rank	Continuous
IMD Decile	Ordered Categorical
Ethnicity	Unordered Categorical
BMI	Continuous
BMI SDS	Continuous
Obese/Non-obese	Ordered Categorical
BMI SDS Change	Continuous
WC	Continuous
WC SDS	Continuous
Body Fat Percentage	Continuous
Self-esteem	Ordered Categorical
Sedentary Behaviour	Continuous
Body Satisfaction	Continuous

Criteria Item #9: *Handling with Non-Normally Distributed and Categorical Data*

All continuous data were parametric and normally distributed. For MI to be completed using the SPSS software, categorical variables needed to be collapsed into binary groups. This resulted in ethnicity and BMI classification being reduced to two categories.

The Fully Conditional specification used in the MI procedure enables multiple data types to be worked with. As such, continuous and categorical (ordered and unordered) variables were used in the MI model.

Criteria Item #10: *Statistical Interaction in the Final Analysis*

There were no statistical interactions in the final analysis.

Criteria Item #11: *Observed and Imputed Values: Sensitivity Analysis of Frequencies and Descriptives*

Variables had a degree of missingness of up to 53.7% (Sedentary Behaviour). Within the guidelines set out by Sterne *et al.* (2009), up to 70% of missingness has shown to be imputed. A study by Fagg *et al.* (2014) which imputed data from a similar source, had missingness of up to 63% in given variables. Data here do not reach this level of missingness, and although there is not a definition of what constitutes as a "large fraction", it would be viable to conclude that 53.7% is a large fraction of data. With that said, the tables below (Table 9 and Table 10) demonstrate the differences between the original data set (*casewise deletion*) and the imputed data set (*imputed*).

Table 9 and Table 10 highlight the mean/percentage values of variables by casewise deletion (whereby *n* varies) and by imputation (of which *n* is the pooled value of 10 data sets). As shown, the values of both the imputed and casewise deletion data do not differ greatly. Where missingness in the variable of interest was relatively low (e.g. BMI SDS, WC SDS) the mean value remained unaltered, although the confidence intervals adjusted slightly. Imputed data appear not to impact the mean/percentage values greatly, and instead are able to retain the original sample size. The proceeding criteria items assess the use of imputed data in the main study analysis.

Table 9: Sensitivity Analysis of Continuous Variables Post Imputation

Variable	Casewise Deletion			Imputed (Pooled)		% Imputed
	Mean	*95% CI*	*n*	*Mean*	*95% CI*	
BMI	26.00	(25.79, 26.21)	2875	25.99	(25.79, 26.20)	2.48%
BMI SDS	2.48	(2.45, 2.52)	2875	2.48	(2.46, 2.51)	2.48%
WC	83.31	(82.77, 83.86)	2602	83.35	(82.84, 83.87)	11.74%
WC SDS	2.93	(2.90, 2.97)	2586	2.93	(2.90, 2.97)	12.28%
Body Fat Percentage	35.21	(34.88, 35.53)	2573	35.15	(34.85, 35.45)	12.72%
IMD Score	30.29	(29.69, 30.88)	2820	30.26	(29.69, 30.83)	4.34%
Self-esteem	4.11	(4.07, 4.16)	2223	4.12	(4.07, 4.16)	24.59%
Sedentary Behaviour	3.46	(3.38, 3.55)	1694	3.60	(3.53, 3.66)	42.54%
Body Satisfaction	28.96	(28.19, 29.72)	2412	28.74	(28.05, 29.43)	18.18%

Table 10: Sensitivity Analysis of Categorical Variables Post Imputation

Variable	Casewise Deletion		Imputed (Pooled)		% Imputed
	%	n	%	n	
IMD Decile					4.34%
1 – Least Deprived	2.27	64	2.17	64	
2	3.58	101	3.43	101.3	
3	4.57	129	4.45	131.4	
4	7.02	198	6.92	204.2	
5	6.28	177	6.43	189.7	
6	9.54	269	9.93	293.1	
7	13.19	372	13.71	404.6	
8	15.35	433	15.53	458.4	
9	19.72	556	19.54	576.7	
10 – Most Deprived	18.48	521	17.91	528.6	
Ethnicity					34.43%
White/White British	67.70	1308	67.14	1981.9	
Non-white/Non-white British	32.30	625	32.86	970.1	
BMI Classification					2.48%
Obese	73.70	2120	73.15	2159.3	
Non-obese	26.30	755	26.85	792.7	

Criteria Item #12: *Results from Complete Case Analysis for Comparison against Imputed Data: Sensitivity Analysis*

The sensitivity analysis which was implemented displays the results of the imputed data and complete case data analyses. This was conducted for participant characteristics, Change in BMI SDS amongst the Completion Status' and finally in the multivariable regression models. All complete case analyses had 906 participants, whereas the imputed analyses made use of 2948 participants. As aforementioned, if analyses were constrained to the complete case analyses this would only account for 30.7% of the sample. Any conclusions or results drawn from complete case analyses may not accurately portray the outcomes of the complete sample.

Table 11 expresses the participant characteristics by imputed data and by complete case data. As can be seen, there are only slight differences between the two data sets in all variables besides Ethnicity. Should analyses have been conducted using complete cases, White/White British participants would have been greatly underestimated in any future analyses.

However, when investigating the impact of missing data on a Change in BMI SDS by Completion Status (Table 12), one can observe that the average reduction amongst the total group would have been greater in the complete case analysis. Imputed data analyses found that mean reduction in

BMI SDS as a result of the programme was 0.10±0.21 units, yet by complete case analysis this would have been a reduction of 0.12±0.07 units. The same is true for those completing the programme: complete case and imputed analyses resulted in a reduction of 0.17±0.21 and 0.15±0.22 units respectively. Although the magnitude of these differences appears not to be large, to a weight management interventionist these differences would be significant.

The final sensitivity analysis assessed the differences between imputed and complete cases in the multivariable regression analyses. Table 13 exhibits the differences in the Odds Ratios (OR) and 95% Confidence Intervals (95% CI). The direction of the OR remained fairly consistent, apart from a number of instances where the OR in the imputed model was very close to 1. The magnitude of the differences in these few instances was marginal. Although the OR continued to be in the same direction, the magnitude of the OR were both inflated and reduced in the complete case analysis. In addition, the 95% CI tended to be larger in the complete case analysis – thus signifying a weaker precision of the actual estimate itself. For example, non-white participants had a 1.56 (95% CI: 1.15, 2.12) greater likelihood of being a Sporadic Attender as opposed to a programme completer in model 4. In complete case analyses, Non-white participants had only 1.32 (95% CI: 0.93, 1.88) times increased likelihood of being a Sporadic Attender, this was also now insignificant (*p* >0.05).

Table 11: Imputed and Complete Case Participant Characteristics

**Characteristic**	**Imputed**		**Complete Case**		**Difference**
	Mean or n	*SD or %*	*Mean or n*	*SD or %*	
Gender [n, %]					
Male	1340	45.45%	388	42.80%	-2.65%
Female	1608	54.55%	518	57.20%	2.65%
Age (Years) [mean, SD]	10.44	2.80	10.21	2.79	-0.01
Ethnicity [n, %]					
White/White British	2079	70.52%	480	53.00%	-17.52%
Non-white/Non-white British	869	29.48%	426	47.00%	17.52%
IMD Score [mean, SD]	30.26	15.90	28.69	14.05	-1.85
IMD Decile [n, %]					
1 – *Least Deprived*	64	2.17%	24	2.60%	0.43%
2	101	3.43%	30	3.30%	-0.13%
3	129.8	4.40%	30	3.30%	-1.10%
4	201.3	6.83%	72	7.90%	1.07%
5	190	6.45%	59	6.50%	0.05%
6	293.2	9.95%	89	9.80%	-0.15%
7	406.6	13.79%	117	12.90%	-0.89%
8	464.3	15.75%	167	18.40%	2.65%
9	573.1	19.44%	203	22.40%	2.96%
10 – *Most Deprived*	524.7	17.80%	115	12.70%	-5.10%

Medical Condition [n, %]					
No	2727	92.50%	852	94.00%	1.50%
Yes	221	7.50%	54	6.00%	-1.50%
BMI (kg/m^2) [mean, SD]	25.99	5.79	25.64	5.83	0.04
BMI SDS [mean, SD]	2.48	0.89	2.44	0.9	0.01
Waist Circumference (cm) [mean, SD]	83.40	15.01	82.35	13.92	-1.09
WC SDS [mean, SD]	2.94	0.97	2.94	0.86	-0.11
Obese or Non-obese [n, %]					
Obese	2161	73.30%	654	72.19%	-1.12%
Non-obese	787	26.70%	261	28.81%	2.11%

Table 12: Imputed and Complete Case Change in BMI SDS by Completion Status

Completion Status	Imputed			Complete			Difference
	n	*Mean*	*SD*	*n*	*Mean*	*SD*	
Completer	1387	-0.15	0.22	462	-0.17	0.21	-0.02
Initiator	548	-0.02	0.20	111	-0.01	0.25	0.01
Non-Completer	380	-0.07	0.21	114	-0.08	0.23	-0.02
High Infrequent Attender	346	-0.09	0.18	121	-0.09	0.02	-0.01
Low Infrequent Attender	287	-0.07	0.18	98	-0.06	0.02	0.00
Total	2948	-0.10	0.21	906	-0.12	0.07	-0.02

Table 13: Imputed and Complete Case Multivariable Models

	Multivariable Model Results (Imputed)			Multivariable Model Results (Complete)		
	OR	*LBCI*	*UBCI*	*OR*	*LBCI*	*UBCI*
**Model 1:** *Completer vs. Non Completer*						
Constant	0.398	0.278	0.571	0.313		
Ethnicity†	1.028	0.835	1.264	0.943	0.709	1.253
IMD Score	1.005	1.000	1.010	0.996	0.986	1.005
BMI SDS	1.111	1.020	1.211	1.184	1.019	1.375
Intervention Year	1.130	1.066	1.199	1.161	1.015	1.327
Group Size†	1.207	1.028	1.417	1.312	0.941	1.828
Delivery Period†						
January Intake			*Reference Category*			
April Intake	1.284	1.077	1.530	1.327	0.975	1.805
September Intake	1.261	1.046	1.520	1.336	0.916	1.949
**Model 2:** *Continuer vs. Initiator*						
Constant	0.079	0.049	0.127	0.069		
Ethnicity†	0.637	0.492	0.825	0.560	0.360	0.870
IMD Score	1.003	0.997	1.009	0.997	0.982	1.011
BMI SDS	1.091	0.976	1.220	1.249	0.988	1.579
Intervention Year	1.178	1.090	1.272	1.072	0.879	1.308
Group Size†	1.399	1.141	1.714	1.428	0.856	2.382
Delivery Period†						
January Intake			*Reference Category*			
April Intake	1.318	1.054	1.648	1.034	0.652	1.638
September Intake	1.363	1.069	1.739	1.049	0.584	1.886

**Model 3:** *Completer vs. Late Dropout*						
Constant	0.097	0.054	0.173	0.141		
Ethnicity†	0.823	0.603	1.125	0.789	0.504	1.236
IMD Score	1.002	0.994	1.009	0.986	0.971	1.002
BMI SDS	1.178	1.023	1.357	1.178	0.925	1.501
Intervention Year	1.167	1.066	1.278	1.156	0.942	1.418
Group Size†	0.957	0.743	1.232	1.068	0.633	1.803
Delivery Period†						
January Intake			*Reference Category*			
April Intake	1.397	1.064	1.834	1.449	0.908	2.312
September Intake	1.180	0.874	1.594	0.774	0.404	1.482
**Model 4:** *Completer vs. Sporadic Attender*						
Constant	0.214	0.134	0.343	0.120		
Ethnicity†	1.565	1.153	2.124	1.320	0.925	1.884
IMD Score	1.006	1.000	1.013	1.000	0.988	1.012
BMI SDS	1.065	0.949	1.195	1.126	0.933	1.359
Intervention Year	1.032	0.954	1.117	1.159	0.979	1.372
Group Size†	1.196	0.970	1.474	1.312	0.867	1.984
Delivery Period†						
January Intake			*Reference Category*			
April Intake	1.123	0.891	1.414	1.309	0.888	1.929
September Intake	1.165	0.913	1.485	1.761	1.114	2.784
**Model 5:** *High vs. Low Sporadic Attender*						
Constant	0.510	0.228	1.144	0.262		
Ethnicity†	1.539	0.980	2.419	1.827	0.984	3.394
IMD Score	1.010	1.000	1.021	1.018	0.998	1.039
BMI SDS	0.863	0.718	1.038	0.981	0.722	1.333
Intervention Year	1.104	0.956	1.275	1.081	0.774	1.510
Group Size†	1.358	0.954	1.933	1.163	0.564	2.398
Delivery Period†						
January Intake			*Reference Category*			
April Intake	0.763	0.517	1.125	0.778	0.404	1.498
September Intake	0.880	0.583	1.330	0.976	0.456	2.093

† Categorical variables
LBCI: Lower Boundary of the 95% Confidence Interval
UBCI: Upper Boundary of the 95% Confidence Interval
Model 1: Imputed (*n* = 1387 vs. 1561), Complete (*n* = 462 vs. 444)
Model 2: Imputed (*n* = 2400 vs. 548), Complete (*n* = 795 vs. 111)
Model 3: Imputed (*n* = 1387 vs. 380), Complete (*n* = 462 vs. 219)
Model 4: Imputed (*n* = 1387 vs. 633), Complete (*n* = 462 vs. 219)
Model 5: Imputed (*n* = 346 vs. 287), Complete (*n* = 121 vs. 98)

Criteria Item #13: *Do the Variables included in the Imputation Model Make the MAR Assumption Plausible?*

The variables in included in the imputation model would make the MAR assumption plausible. As detailed previously (*Criteria Item #2*), data were missing due to programme managers not collecting all available data – some chose not to assess Sedentary Behaviour for example. In addition, one of

the completion groups, Non-Initiators, were responsible for a large proportion of missing data. These participants did not attend any of the MoreLife sessions and this resulted in no data being collected for this completion group. The outcome variable, alongside a total of 20 others, were included in the imputation model meaning that the model accounted for each of the included variables when imputing missing data. We cannot ever know what the true missing values are, however the process of MI ensures that the imputed values have the greatest likelihood of being a close-to-accurate estimate. As discussed, data were not MCAR due to the outcome of a series of Little's MCAR tests. The missingness was assumed to occur, and could be explained, by the observed variables and data.

Criteria Item #14: *Investigate Robustness of Key Inferences to Possible Departures from the MAR Assumption – Assume a Range of MNAR Mechanisms in the Sensitivity Analysis.*

Should missing data be assumed to be MNAR, then the missingness mechanism would infer that data are missing due to either one or more latent variables. In other words, the unobserved variables are responsible for missing data. There is a strong case to believe data are MAR in this study, and the research team/MoreLife team could not theorise another possibility for why data are missing. We conclude that, to the best of our knowledge and expertise, data were solely missing due to the mechanisms explained: Programme Commissioning and Non-Initiation.

References:

1. White IR, Royston P, Wood AM. Multiple imputation using chained equations: issues and guidance for practice. *Stat Med.* 2011;**30**: 377-399.

2. Sterne JAC, White IR, Carlin JB, *et al.* Multiple imputation for missing data in epidemiological and clinical research: potential and pitfalls. *BMJ.* 2009;**338**.

3. Fagg J, Cole TJ, Cummins S, *et al*. After the RCT: Who comes to a family-based intervention for childhood overweight or obesity when it is implemented at scale in the community? *J Epidemiol & Comm Health.* 2014.

4. Foster P, Mami A, Bala A. *On treatment of the multivariate missing data*. School of Mathematics: University of Manchester, 2009. 48.

5. Schafer JL. Multiple imputation: a primer. *Stat Methods Med Res.* 1999;**8**: 3-15.

6. Little RJA, Rubin DB. *Statistical analysis with missing data*. Wiley, 1987.

7. Hayati Rezvan P, Lee KJ, Simpson JA. The rise of multiple imputation: a review of the reporting and implementation of the method in medical research. *BMC Med Res Methodol.* 2015;**15**: 30.

8. Rubin DB. *Multiple imputation for nonresponse in surveys.* Wiley, 2004.

9. van Hoek E, Feskens EJ, Bouwman LI, Janse AJ. Effective interventions in overweight or obese young children: systematic review and meta-analysis. *Child Obes.* 2014;**10**: 448-460.
