# Supplementary material for: Design programmes to maximise participant engagement: a predictive study of programme and participant characteristics associated with engagement in paediatric weight management
Source: Int J Behav Nutr Phys Act. 2016 Jul 19;13:76. doi: 10.1186/s12966-016-0399-1 (PMC4949908; doi:10.1186/s12966-016-0399-1)
Supplement: Additional file 3: — Univariable Regression Models. (PDF 411 kb) [file 12966_2016_399_MOESM3_ESM.pdf]

<u>Additional File 3</u>: *Univariable Regression Models*

The tables below provide the results of all univariable regression models. Each variable was entered into the regression independently alongside the outcome variable. These tables should be used ancillary to the main results table (Table 4) in the paper.

Model One (Completers vs. Non-Completers)

**Variable**		**Bivariate Results**			
	β - value	OR	LBCI	UBCI	*p*-value
Gender†	-0.079	0.924	0.799	1.068	0.28
Age	-0.015	0.985	0.960	1.011	0.26
Ethnicity†	0.150	1.162	0.962	1.402	0.12
IMD Score	0.005	1.005	1.000	1.010	0.03
Medical Condition†	0.078	1.081	0.821	1.424	0.58
BMI SDS	0.073	1.076	0.990	1.170	0.09
WC SDS	0.101	1.107	1.018	1.203	0.02
Obese or Non-obese †	0.065	1.067	0.903	1.261	0.45
Self-esteem	-0.039	0.962	0.893	1.036	0.30
Sedentary Behaviour	0.039	1.040	0.990	1.092	0.12
Body Satisfaction	-0.002	0.998	0.994	1.002	0.38
Programme Length†	0.011	1.011	0.873	1.172	0.88
Programme Year	0.122	1.130	1.071	1.192	0.00
Group Size†	0.230	1.259	1.087	1.457	0.00
Age Groups†	-0.013	0.987	0.864	0.853	1.14
Day of Session Delivery†	0.052	1.054	0.908	1.223	0.49
Delivery Period†					
January Intake		*Reference Category*			
April Intake	0.218	1.244	1.046	1.479	0.01
September Intake	0.075	1.078	0.904	1.287	0.40

†Categorical variables (dummy coded [0 or 1])
LBCI: Lower Boundary of the 95% Confidence Interval
UBCI: Upper Boundary of the 95% Confidence Interval

Model Two (Continuers vs. Initiators)

**Variable**		**Bivariate Results**			
	β - value	OR	LBCI	UBCI	*p*-value
Gender†	0.001	1.001	0.831	1.206	0.99
Age	0.010	1.010	0.977	1.044	0.56
Ethnicity†	-0.245	0.783	0.617	0.992	0.04
IMD Score	0.004	1.004	0.998	1.010	0.21
Medical Condition†	0.123	1.130	0.804	1.591	0.48
BMI SDS	0.070	1.073	0.962	1.196	0.21
WC SDS	0.090	1.094	0.979	1.222	0.11
Obese or Non-obese†	0.102	1.108	0.887	1.383	0.37
Self-esteem	0.014	1.014	0.928	1.109	0.75
Sedentary Behaviour	0.040	1.041	0.973	1.114	0.24
Body Satisfaction	-0.001	0.999	0.994	1.005	0.80
Programme Length†	0.051	1.052	0.870	1.272	0.60

Programme Year	0.133	1.143	1.065	1.226	0.00
Group Size†	0.282	1.326	1.101	1.598	0.00
Age Groups†	0.007	1.007	0.835	1.213	0.95
Day of Session Delivery†	0.043	1.044	0.863	1.264	0.66
Delivery Period†					
January Intake		*Reference Category*			
April Intake	0.216	1.242	0.996	1.548	0.05
September Intake	0.095	1.100	0.874	1.384	0.42

†Categorical variables (dummy coded [0 or 1])
LBCI: Lower Boundary of the 95% Confidence Interval
UBCI: Upper Boundary of the 95% Confidence Interval

Model Three (Completer vs. Late Dropout)

**Variable**		**Bivariate Results**			
	β - value	OR	LBCI	UBCI	*p*-value
Gender†	-0.066	0.936	0.745	1.175	0.57
Age	-0.016	0.985	0.944	1.026	0.47
Ethnicity†	-0.124	0.883	0.661	1.181	0.40
IMD Score	0.002	1.002	0.994	1.009	0.65
Medical Condition†	0.235	1.265	0.842	1.900	0.26
BMI SDS	0.155	1.168	1.017	1.341	0.03
WC SDS	0.196	1.216	1.060	1.395	0.01
Obese or Non-obese†	-0.018	0.982	0.756	1.276	0.89
Self-esteem	-0.061	0.941	0.840	1.053	0.29
Sedentary Behaviour	0.060	1.062	0.988	1.141	0.10
Body Satisfaction	0.001	1.001	0.994	1.008	0.77
Programme Length†	0.060	1.061	0.841	1.339	0.62
Programme Year	0.116	1.123	1.033	1.222	0.01
Group Size†	-0.032	0.969	0.768	1.222	0.79
Age Groups†	-0.042	0.959	0.762	1.205	0.72
Day of Session Delivery†	0.085	1.089	0.863	1.374	0.47
Delivery Period†					
January Intake		*Reference Category*			
April Intake	0.290	1.336	1.022	1.746	0.03
September Intake	0.039	1.040	0.784	1.380	0.79

†Categorical variables (dummy coded [0 or 1])
LBCI: Lower Boundary of the 95% Confidence Interval
UBCI: Upper Boundary of the 95% Confidence Interval

Model Four (Completer vs. Sporadic Attender)

**Variable**		**Bivariate Results**			
	β - value	OR	LBCI	UBCI	*p*-value
Gender†	-0.120	0.887	0.734	1.071	0.21
Age	-0.029	0.971	0.939	1.005	0.10
Ethnicity†	0.505	1.656	1.264	2.171	0.00
IMD Score	0.007	1.007	1.001	1.013	0.03
Medical Condition†	-0.089	0.915	0.630	1.329	0.64
BMI SDS	0.012	1.012	0.906	1.130	0.84

WC SDS	0.031	1.031	0.923	1.152	0.59
Obese or Non-obese†	0.067	1.069	0.859	1.330	0.55
Self-esteem	-0.049	0.952	0.868	1.044	0.29
Sedentary Behaviour	0.013	1.013	0.953	1.078	0.67
Body Satisfaction	-0.004	0.996	0.991	1.002	0.17
Programme Length†	-0.049	0.953	0.787	1.153	0.62
Programme Year	0.082	1.085	1.012	1.163	0.02
Group Size†	0.279	1.322	1.094	1.597	0.00
Age Groups†	-0.005	0.995	0.823	1.203	0.96
Day of Session Delivery†	0.024	1.024	0.843	1.243	0.81
Delivery Period†					
January Intake		*Reference Category*			
April Intake	0.109	1.115	0.890	1.398	0.35
September Intake	0.061	1.063	0.846	1.336	0.60

†Categorical variables (dummy coded [0 or 1])

LBCI: Lower Boundary of the 95% Confidence Interval

UBCI: Upper Boundary of the 95% Confidence Interval

Model Five (High vs. Low Sporadic Attender)

**Variable**		**Bivariate Results**			
	β - value	OR	LBCI	UBCI	*p*-value
Gender†	0.026	1.026	0.750	1.404	0.87
Age	-0.063	0.939	0.888	0.993	0.02
Ethnicity†	0.649	1.914	1.304	2.811	0.00
IMD Score	0.010	1.010	1.000	1.021	0.05
Medical Condition†	-0.004	0.996	0.531	1.867	0.99
BMI SDS	-0.226	0.789	0.668	0.952	0.01
WC SDS	-0.120	0.887	0.745	1.056	0.18
Obese or Non-obese†	0.550	1.733	1.212	2.479	0.00
Self-esteem	0.028	1.029	0.884	1.197	0.71
Sedentary Behaviour	0.062	1.064	0.962	1.178	0.23
Body Satisfaction	-0.002	0.998	0.990	1.007	0.70
Programme Length†	-1.089	0.336	0.242	0.467	0.00
Programme Year	0.207	1.230	1.089	1.389	0.00
Group Size†	0.537	1.711	1.247	2.346	0.00
Age Groups†	0.194	1.214	0.886	1.664	0.23
Day of Session Delivery†	-0.421	0.657	0.474	0.910	0.01
Delivery Period†					
January Intake		*Reference Category*			
April Intake	-0.313	0.731	0.502	1.066	0.10
September Intake	-0.286	0.751	0.513	1.100	0.14

†Categorical variables (dummy coded [0 or 1])

LBCI: Lower Boundary of the 95% Confidence Interval

UBCI: Upper Boundary of the 95% Confidence Interval
